# Application of the *Food Guide for the Brazilian Population* as a training instrument for intersectoral actions: perceptions of professionals in a Brazilian metropolis

**DOI:** 10.1590/S2237-96222025v34e20240397.en

**Published:** 2025-05-23

**Authors:** Vanessa Fernandes Davies, Luiza Navarro de Azevedo, Roberta Camila de Lima Pinheiro, Larissa Galastri Baraldi

**Affiliations:** 1 Universidade Estadual de Campinas, Núcleo de Estudos e Pesquisas em Alimentação, Campinas, SP, Brazil; 2 Universidade de São Paulo, Faculdade de Saúde Pública, São Paulo, SP, Brazil

**Keywords:** Intersectoral Collaboration, Food Guides, Interview, Professional Training, Children, Colaboración Intersectorial, Guías Alimentarias, Entrevista, Capacitación Profesional

## Abstract

**Objective:**

To analyze perceptions arising from an educational intervention based on the *Food Guide for the Brazilian Population*, aimed at health, education and social services professionals who work with children.

**Methods:**

This was a qualitative study conducted with nine professional training course participants, using semi-structured interviews. The interviews took place one year after the training, on an online platform, with average duration of 30 minutes. The interview was validated previously, including questions to explore perceptions about the training course, the applicability of the Food Guide and challenges faced. Thematic analysis was performed in stages: familiarization with the data, direct labeling, data extraction and organization into matrices, development of preliminary themes and review to define final themes.

**Results:**

The interviews were conducted with nine female professionals. Based on analysis of the data, the following themes were defined: Training and professional enhancement; Reflections produced by the Guide; and Intersectoral integration and collaboration.

**Conclusions:**

*The Food Guide for the Brazilian Population* promoted reflections on challenges in implementing actions to prevent childhood obesity.

## Resumen

**Objetivo:**

Analizar las percepciones que surgieron de una intervención educativa basada en la *Guía Alimentaria para la Población Brasileña*, dirigida a servidores públicos que actúan con la población infantil.

**Métodos:**

Estudio cualitativo realizado con nueve participantes de una capacitación profesional, mediante entrevistas semiestructuradas. Las entrevistas se realizaron un año después de la capacitación, en una plataforma online, con una duración promedio de 30 minutos. La entrevista fue previamente validada, incluyendo preguntas para explorar percepciones sobre la capacitación, la aplicabilidad de la Guía y los desafíos enfrentados. El análisis temático se realizó por etapas: familiarización con los datos, etiquetado directo, extracción y organización de datos en matrices, desarrollo de temas preliminares y revisión para definir temas finales.

**Resultados:**

Las entrevistas se realizaron a 9 personas del sexo femenino. A partir del análisis de los datos, se definieron los siguientes temas: Capacitación y enriquecimiento profesional; Reflexiones producidas a partir de la Guía; e Integración y colaboración intersectorial.

**Conclusiones:**

La *Guía Alimentaria para la Población Brasileña* promovió reflexiones sobre los desafíos en la implementación de acciones para prevenir la obesidad infantil.

**Ethical aspects**

Research Ethics Committee: Universidade Estadual de Campinas

Opinion number: 4,924,797

Approval date: 25/8/2021

Certificate of Submission for Ethical Appraisal: 42713521.8.0000.5404

Informed Consent Form: Obtained from all participants before data collection

## Introduction

In the 2010s, global transformations (1) impacted the diet of all populations (2,3), boosting chronic health conditions in all age groups, especially obesity, which challenges the health system (4-6). Analysis of public policies for child health shows that combating overweight and obesity must involve advertising control and agreement with the food industry; by overcoming the obesogenic environment; and by implementing measures that promote healthy school meals, through access to food derived from family farming, as well as food and nutritional education (7). Lack of integrated and intersectoral approaches is a barrier to improving children’s nutritional status, which makes professional training essential for work from this perspective (6-8).

The *Food Guide for the Brazilian Population* is an essential document regarding prevention of obesity, as it values ​​cultural and regional eating practices; it is also an important reference for several areas (9), strengthening intersectoral planning (6,10). Its principles highlight that “Recommendations on food must be in line with their time”, emphasizing the need for a broad view on health and food to mitigate the negative impacts of the current food system in Brazil, aiming for a more sustainable model. In the ten years after it was released, the Guide became a national and international landmark (10,11), proposing a new nutrition paradigm that considers food in its relationship with agri-food systems and culture, beyond the simple intake of nutrients (12-14).

In the case of children and health-related services, the document can guide clinical care, and unfold into specific guidelines for this stage of life (7,15). In the school environment, the contents of the Guide must be integrated into teaching practices and food and nutritional education actions, so as to foster autonomy and critical awareness about food among schoolchildren, becoming a reference for the entire community, including parents and educators (16). The new parameters for purchasing school meals promote purchasing of fresh and minimally processed foods, to the detriment of ultra-processed foods, as they incorporate the Guide’s NOVA food classification and promote a healthier consumption pattern among schoolchildren (17). Furthermore, with regard to intersectoral collaboration between the areas of Education and Health, institutionalized by the National School Meals Program and the National Food and Nutrition Policy, it is essential for them to work together to encourage eating habits that respect diversity and food sovereignty (7,18,19). 

This study is based on the hypothesis that the Guide offers relevant content for various professionals who work with children, and can improve work practices and encourage reflections on factors that affect the promotion of adequate and healthy eating. We sought to analyze the perceptions of health and education professionals involved in an educational intervention on the content of the Guide, focusing on daily practices, facilitators and challenges in implementing actions to prevent childhood obesity and how professionals absorbed the concepts contained in the Guide.

## Methods

### Study type

This was a qualitative study intended to enable understanding of the perceptions and accumulated experiences of the participants, exploring the applicability of the Guide in the training of health and education professionals. Aligned with the study’s hypothesis, objectives and collection technique, its pragmatic theoretical-methodological approach allowed free exploration of the data, with inductive analysis of the statements made during the interviews, in order to arrive at the findings presented (20,21).

### Description of the educational intervention

The educational intervention was part of the Intersectoral Actions for the Prevention of Childhood Obesity project – conducted in partnership between Campinas City Government, the *Universidade Estadual de Campinas* Center for Food Studies and Research and the Brazilian Ministry of Health. Workshops were held, totaling 16 hours of training, divided into four modules, with activities adapted and validated for children and the intersectoral context (22,23). The subjects covered in the workshop were intended for health, education and social assistance professionals, addressing the main contents of the Guide, with focus on preventing childhood obesity and promoting healthy eating. The training activities, carried out between February and March 2022, took place in a hybrid format, with weekly online and in-person meetings, conducted by facilitators and based on critical-reflective methodology (24). The training resulted in a Ministry of Health technical manual for future workshops and included topics such as: centrality of the Guide in food and nutritional education; principles of the Guide and healthy eating; obesity prevention; the NOVA classification; food environment and commensality (the practice of eating together); challenges in promoting child health; and developing of a work plan. Further details on this intervention can be found in Supplementary Figure 1.

### Sample selection and characterization

We used intentional sampling composed of professionals who were present at the training. The number of participants ranged from 27 in the online module to 13 in the last in-person module. All 27 participants were invited to evaluate the intervention via email and telephone, on at least three different occasions, on different days and times. In the end, nine health and early childhood education professionals agreed to be interviewed; three stated they would not be able to take part in the interview; and the remainder did not reply to the invitation.

### Data collection

Semi-structured interviews were conducted in order to collect data. A script was developed, in line with the main research question. Each question in the script had its own objective, aligned with the main research question, ensuring systematization of the information collected and support for the interviewer to explore the question investigated. The interviewer was trained to act as a facilitator, helping the interviewee to understand the questions and feel comfortable expressing their opinion. Additionally, an attitude of acceptance, attention and care was adopted towards the participants, in order to facilitate the fluidity of the interviews.

The script was validated by the project coordinator, an expert, according to the criteria of coherence with the objectives, clarity of questions, relevance of themes and scope (25). The questions followed an increasing sequence of complexity and included follow-up questions to deepen the topics covered. Table 1 shows the interview script in detail. 

**Table 1:** Semi-structured script used in interviews conducted with professionals who participated in the training course. Campinas, 2023

Main question	Follow-up question	Objective of the question	Level of complexity
Tell me what you thought about the training course.	If you were to choose a subject within the training course that you didn’t know and that you found interesting, what would it be? Tell me what you think could be improved in the training course you took part in.	To gain knowledge on the overall opinion about the training course.	Opening question to initiate the relationship between interviewee and interviewer and, at the same time, allow the expression of feelings and opinions about adjustments for future training courses.
Tell me about what you learned during the training course; What did you manage to put into practice?	What did you learn in the training course that you find difficult to put into practice? Why?	To evaluate how the participants imagine that what was taught in the course can be implemented in practice.	Intermediate-level question, which requires the interviewee to reflect on their learning in the training course.
During the training course, the term “commensality” was presented, which is linked to the act of eating in company. Tell me what you think about this concept.	Following the training course, how do you approach commensality, or eating in company, and the promotion of healthy eating?	To understand what was learned about commensality, a key concept in the Food Guide for the Brazilian Population.	Intermediate level question, but a little more complex than the previous one, as it requires the interviewee to seek to learn about an unusual concept in their work reality.
I don’t know if you remember, but people from different areas participated in the training workshops. What did you think of this integration?	How do you see people from different areas working to promote healthy eating? And in your work, how do you imagine different professionals working on the topic of promoting healthy eating? What could be done to integrate people from different areas to work on the topic of healthy eating?	To assess participants’ understanding of intersectoral actions to prevent childhood obesity.	Complex question, as it may involve feelings related to work processes, and work dynamics with professionals from different areas.
Tell me how you perceive integration between health and education professionals for working on promoting healthy eating.	What facilitates this integration taking place? And what are the difficulties for integration taking place? What could be improved?	To evaluate the applicability of what was covered in the training course in relation to the consolidation and dissemination of Health Promotion and Adequate and Healthy Food actions in the intersectoral practices of health and education professionals in their catchment territories.	Complex question, as it involves questions related to opinions, reflections on possibilities and obstacles to be addressed, as well as the construction of solutions to the problem presented.
Finally, I would like to know if there is anything else you would like to say about the training course that was not covered in the questions we asked you.	-	To promote openness, allowing the interviewee to complement or address something relevant not explored in the interview.	Final question, to give the interviewee the opportunity to express themselves.

Contact with the participants occurred one year after the intervention, so as to enable evaluation of the consequences of the intervention and the experiences remembered by the professionals. The interviews, carried out in March 2023, lasted 30 minutes on average and were recorded via Google Meet.

### Data analysis

The interview recordings were uploaded to the PinPoint platform for transcription. A manual review was necessary to ensure the fidelity of the transcriptions, which was carried out word by word, to as ensure maximum accuracy.

Two researchers, trained by a senior researcher, independently conducted thematic analysis of the data. The method chosen allows adaptations to different theoretical approaches, facilitates the synthesis and description of large data sets, identifies similarities and differences between interviewees, generates unexpected insights and enables social and psychological interpretations of the data (26).

The analytical process consisted of the following steps: familiarization with the data, which included reading and re-reading the transcriptions; creation of codes, based on the relevance of the extracts to the research question; direct coding of transcribed interviews (Supplementary Table 1); extraction and organization of data into matrices according to codes (Supplementary Table 2); preliminary analysis for theme development; and subsequent review, to verify the internal and external coherence of the themes, culminating in the definition of the final themes that summarize the content of the interviews (26). A detailed summary of the analysis steps is available in the supplementary material.

**Table 2:** Excerpts extracted from the spoken answers of interviewees participating in the educational intervention, according to the theme created. Campinas, 2023

Theme	Quote
I **Training and professional enhancement**	“...it brought another way of looking at how to use the Guide as a tool... I still remember a chat we were having and it was a brainstorm: “what is food for children?”... and then several people participating – not all from the health area –, one said “Danoninho”, and the other said “Toddynho”... So, to understand how this vision is wrong, but it is already embedded in people, and we end up using it as a reference and this is what we have been trying to change, with this work that is being done, so... Open your eyes to the Food Guide?” Case 6, education professional. “It’s very easy for us to hear about the Food Guide... But many people don’t know about it... But this is work that has to be continuous, not... just once... I think it’s something ... So, “hitting the same key” several times, continuously, so that, yes, we see a difference - sometimes small - but we see a difference, right?” Case 6, education professional. “I really didn’t have an idea of ​​what processed, ultra-processed, minimally processed food was, you know?” Case 5, education professional. “And what you spend on one, you can buy much more on fresh foods, we really have the idea that fresh foods are more expensive than processed foods, than ultra-processed foods, right? I said: so, wait a minute, wow!” Case 5, education professional. “Being in contact with other professionals, also from other places. Not only other professionals from the same health center, but from other places as well. I think... It was worth seeing a little of what was different in each of the places, in each of the sectors and the things that were similar, the difficulties that were similar in that sense.” Case 1, health professional. “I found it very interesting, mainly because it involved professionals from other areas... Other areas, right? But they also work with the public we serve, right? So I found that very interesting. It’s... It was possible for us to exchange ideas, for people to learn more about school food work as well. And the dynamics used, right? The activities used to build actions are... To build understanding of what was explained by the nutritionists, I thought it was very interesting.” Case 4, education professional. “We had the dynamic of cutting and pasting, of thinking about the territory... right? I didn’t know many people there. I... I, in fact, I made contacts there and later developed projects with these people – a lot – from the health center, I was invited to speak about the PNAE [National School Meals Program] to a residency group there at the São Bernardo health center through a contact I made at that meeting.” Case 5, education professional. “Everyone has a point of view, because everyone has an experience. Whether in a health center or a school, each... A different vision, but the theme was the same. So I think this is very important… I think… The connection… And having more meetings like this would make a difference, you know? Because that way we understand part of another side... Because everything is communicated in the end.” Case 6, education professional..
II **Reflections produced by the Food Guide for the Brazilian Population**	“In a cross-cutting manner, in some way, I can use all the content of the Guide... I can use the discussion of food systems, commensality, for collective, community organization, it’s easy to use too... Ah, I think that in relation to the content of the Guide I can use everything.” Case 1, healthcare professional. “... We are very much hostage to the issue of industry, of media, which was also raised in the training, so, TV commercials, advertisements, supermarket stuff... Anywhere we go... Trying to take this and show it to the mothers in that sense.” Case 1, healthcare professional. “So for me, commensality, which is this moment that is often social, also includes this concept of opportunity. To work on food and nutritional education and improve the quality, lifestyle, and eating habits of children. And then, this will be progressive for older students and then for secondary students. So, they... I think so, that they... They have been in the health network since the beginning. So, if we already have this with the little ones, we will end up with a teenager who eats well, and then an adult who has good habits... That’s the concept I have.” Case 3, healthcare professional. “Well... I cover three, four health centers, how much time do I have to spend with my work colleagues? How much time do I have to build knowledge together? Between traveling, between an agenda of providing a thousand things, so, you have to have a work proposal drawn up for these meetings to happen.” Case 1, health professional. “I think it is very important in the sense that we can, precisely through this concept, raise awareness that our problem ‘is much more under the surface’. So, because we focus a lot, exactly on the child, on eating in company, stimulation, but the examples that the child has are not positive examples and are not healthy examples, so it is a basis of having to take this as an important concept, precisely for the family to understand this, that the family, the friends, the school wherever it is, there is no point in us guiding, doing everything... Putting the child to eat together, doing everything and then wanting the child to eat healthily, if the whole family, if the whole, a school environment, is not conducive to that, it’s not favoring the child following an example.” Case 1, healthcare professional. “I always seek to address this issue of the importance of eating at the table with the family... Talk about the food that is being served, the example that the adult is and the security that he or she provides the child with; and we realize, by the parents’ faces, that when we say that the child has to eat at the table and not in front of the TV or the computer, the reaction is that how does this happen at home. That they don’t eat meals at the table, they don’t eat them together. And at school, we note that when children eat together, one ends up imitating the other. If one doesn’t want to, the other also says he doesn’t want to and the educator insists that he tastes it; one of them tastes it, then the other ends up tasting it too… So, this getting together, this moment of the meal, is extremely important.” Case 4, education professional. “I am someone who likes to cook, I cook every day, I don’t buy anything from outside my home, I like to cook. So, it makes a difference, but all of this, I think it was a seed planted there when I was a child.” Case 6, education professional. “...Because, apart from the Food Guide, we have the Brazilian Society of Pediatrics manual, which we also use and which pediatricians use, more as a reference than the Guide itself. But they… They are well aligned. So, there is another thing, which we, unfortunately, see is a little targeted, because there is sponsorship involved.” Case 8, healthcare professional.
**III** **Intersectoral integration and collaboration**	“If there hadn’t been this facilitating space, we wouldn’t have really accessed each other, I think it would be very difficult, I think there needs to be a person responsible for promoting this meeting, promoting this integration, with these professionals; so, a person who is there in contact with all the services, who brings together these professionals who would be willing to carry on with actions like this. If there hadn’t been one, we wouldn’t have the means to do this. I think there might even happen, but you would have to appoint someone responsible for doing this and maybe it would take a lot longer.” Case 2, education professional. “We see the need for public policies to be planned together, to be an integrated public policy plan. Challenging, very challenging; this is also reflected on the front line. But it’s a discussion that we talk about a lot, because the service user is a whole person, he is not a fragmented person. So why do we also fragment all services?” Case 3, healthcare professional.

After the independent analysis, the researchers met to compare and consolidate the analyses, discussing the final grouping of themes. Disagreements were debated between the authors of this study, in order to reach consensus. The complete database, with all themes and labels created, is available in a virtual data repository (27).

## Results

All nine respondents were female, 43 years old on average and had been working as civil servants for 11 years on average. Five were nutritionists (one working as part of a team at the Family Health Support Center and four working with school meals), a pediatrician, a sociologist working in school vegetable garden projects, an occupational therapist working as part of a team at the Support Center for Family Health and a headmistress of a municipal children’s school. 

Based on data analysis, the following thematic areas were defined: Training and professional enhancement; Reflections produced by the Guide; and Intersectoral integration and collaboration.

Presented below are details of the themes arising from the analyses of the interviews. Details with examples of the interviewees’ statements within each theme can be found in Table 2.

With regard to theme I, participants pointed to the participatory methodology used during training as a positive factor. Likewise, the Guide as a theoretical reference for training was cited as a positive point. Lack of knowledge of the material was reported, not only on the part of professionals, but also on the part of the general population. Participants expressed concern about some products for children widely used in everyday life, considered “children’s food”, but which, in reality, are ultra-processed; the feeling expressed pointed out how the Guide’s recommendations, in practice, had not yet been assimilated by the workshop participants. Based on the workshops, the need was identified to disseminate materials contained in the Guide and the contents that attracted the most positive attention and added theoretical content, according to the professionals, within the material used were listed, namely: the NOVA food classification; the cost of fresh foods compared to ultra-processed foods; application of the concept of commensality; development of culinary skills; and the importance of nutrition labeling.

The other comments in favor of the training course were related to the relevance of the type of integration it generated, as group activities made it possible to compare different technical-professional points of view, making it possible to envision different ways of applying the knowledge acquired. The professionals reported that gaining knowledge of the experiences and difficulties of different workplaces, within the same municipality, makes it possible to plan how to address them, whether jointly in the territory or locally. The need for permanent adoption of diverse training strategies that involve, for example, a better understanding of the realities and work contexts of other municipal employees was highlighted. This set of collaborative practices was seen as essential for reaching a wider audience, including parents, educators and the territory’s community, aiming to promote a comprehensive approach to child health.

Some unfavorable aspects of the training course were mentioned, such as the need for more time for the workshops, the lack of depth in some topics, and the low level of adherence of professionals to the training course, especially education employees. 

The reflections produced by the interviewees in theme II probably originated as a result of the main theoretical framework used, i.e. the Guide. Some interviewees related aspects of the Guide to their personal experiences and professional practice. For example, some participants pointed out that they have the habit of cooking their own food, a fact that, in turn, relates to the point covered in the Guide on culinary skills; the importance of being a critical individual in the face of food industry advertising; and the understanding of the concept of commensality to promote healthy eating habits. 

The professionals also pointed out contextual issues, such as the relevance of food environments in the formation and determination of eating habits. They discussed mainly the dissimilarity between the home and school food environment, considering the variety of school meals, which are often not repeated at home due to the lack of access to different foods. Family influence in establishing eating habits was highlighted, with the active participation of the family during meals being positively highlighted, with the creation of an environment conducive to trying new foods. 

Commensality was pointed out as an agent of direct inference at meal times and its reverberations. The majority of interviewees reported that they always addressed this topic in their daily work, although some did not use the term “commensality” or were not aware, prior to training, of the magnitude of the concept of commensality. “Eating in company” was suggested as a term for promoting “healthy eating” and the introduction of new foods for children, in line with the practice of “pedagogical tasting” during the school meal period, which is the proposal that educators should eat together with students as a form of food and nutrition education. 

The interviewees were open to using the Guide in their work processes. However, they reported the existence of other commonly used materials, such as manuals organized by medical societies. The possibility of conflict of interests, found in materials from different societies, was contradictorily mentioned by professionals who are accustomed to using them. 

The importance of intersectoral integration and collaboration in promoting adequate and healthy nutrition was explored in theme III, with emphasis on the unique contribution of each sector to a more comprehensive approach to child care. Health and education professionals unanimously recognized the need for mutual cooperation to achieve more effective results. Assertive communication and regular meetings between multidisciplinary teams were factors identified as fundamental for the success of the initiatives.

The participants expressed the importance of intersectorality in their daily work, given that public policies are planned from this perspective. Following the same logic, it was mentioned that the public they attend to cannot be treated in a fragmented way, thus bringing the perspective of comprehensive care.

As a positive result of the training course, the interviewees reported progress in communication between professionals. In particular, the creation a WhatsApp group between the training course participants can be highlighted, which was understood as facilitating communication, making it more constant and organized among these health and education professionals. It is important to highlight this improvement, given that, prior to the training, these employees – although they worked in the same region of the municipality – did not know each other and did not have an efficient channel for dialogue. Implementation of this communication tool contributed to strengthening the professional network and improving contact between the group, which allowed the organization of new meetings not involving training, becoming a potential inducer of food and nutrition actions among these employees. The importance of a space (like the one provided during the training) and a leader to organize moments of professional interaction was mentioned as something that would facilitate exchanges and activities to promote adequate and healthy eating, given the great demands that professionals already face in their daily work.

The challenges in implementing intersectoral actions were made evident by the lack of communication between sectors, work overload in small teams, resistance to changes necessary for food and nutrition education, in addition to limitations in adequate and committed human resources. These obstacles, according to the interviewees, make it difficult to build collaborative relationships and carry out joint actions, impacting the effectiveness of health interventions and disease prevention. Persistence and continuity of actions were identified as essential for addressing the aforementioned challenges.

Despite the challenges mentioned, an intersectoral approach was recognized as essential for providing comprehensive care and understanding patients’ needs more broadly. In summary, intersectoral collaboration emerged as a crucial approach to addressing challenges in promoting health and healthy eating, demanding commitment, persistence and an adequate allocation of resources in order for it to be successful.

## Discussion

The educational intervention based on the Guide was well received and there was receptiveness to using the guidelines contained in it, so as to share work practices and new opportunities for intersectoral interaction. 

Implementation of the Guide contributes to this form of organization by promoting a comprehensive health approach, aiming to change eating habits and reduce consumption of ultra-processed foods, which can help prevent and control obesity (28,29). Distributing educational materials and training facilitators are the most common ways of implementing food guides, which can positively impact the demand for public policies, to the extent that the recommendations being adopted by the population enhances the overcoming of systemic obstacles. However, putting this process into practice requires education and health sector engagement, which often proves challenging, due to the lack of alignment of agendas, knowledge or interest, as evidenced in this study (29). The experience of the Guide’s applicability in training professionals in two key areas for promoting adequate and healthy eating is a contribution of this study. Encouraging reflections on topics not covered in the traditional training of professionals, other than nutritionists, can provide an increase in individual and collective confidence as to the practical application of the content covered in the training course (30). Part of the acceptance of adopting the Guide as an official document for supporting professional practices is due to the fact that it has proven to be a multidisciplinary support material, which dialogues with different aspects of nutrition that are part of the daily lives of all individuals, which aroused the interest of the participants. This corroborates the statement that the Brazilian Food Guide meets the objectives and goals expected of a quality food guide, being material based on scientific evidence, practical, easy to understand and adaptable to different age groups (31,32).

The reflections produced among the participants regarding commensality were a positive finding, since the aspects covered in this dimension – ranging from the way of eating and having company at the table, to the time and attention dedicated to meals – can be considered decisive for a healthy diet (33). Adoption of the concept of commensality is one of the innovative points of the Guide and contributes to the change from nutrition-centered and reductionist approaches to a more holistic and sustainable way of approaching food (34,35). We found that the different dimensions of the Guide, especially those that do not directly address dietary and nutritional recommendations, are those that most bring professionals who do not have training in nutrition closer together.

Although the training course participants demonstrated willingness to integrate the Guide’s content into their work processes, an alert was raised by one of the participants regarding the tendency of health professionals to use reference materials from organizations and societies from different medical specialties, which may be biased due to partnerships with companies with conflicts of interest. For future training, a more direct comparison between these frequently produced materials and the content of the Guide is suggested, which could encourage critical reflections on the influence of the food and pharmaceutical industries on the development of materials and health practices. Conflict of interest has been widely discussed in the relationship between academics and funding of production of scientific evidence, but may not be a fully recognized concern among health professionals (36). The use of the Guide to address this issue is relevant, as it is a resource based on scientific evidence and free from economic interests.

The creation of a permanent communication group, through the WhatsApp application, to exchange experiences between the participants in this study, may have been facilitated by the methodology used in the workshops, which favored rapprochement between the professionals. The dialogical approach, particularly that of Paulo Freire, encourages dialogue and critical reflection, promoting an environment conducive to building bonds, forming networks and continuous collaboration between educators (25). The group formed constituted a first step towards overcoming intersectoral barriers, considering the geographical divisions in large cities, such as Campinas, and the lack of means that allow communication between professionals from different areas. More research is needed on the role of technologies, such as cell phones, in facilitating professional collaboration, overcoming limitations of physical access and time (37). As highlighted by the interviewees, investment in financial and human resources is essential for sustaining and maximizing the benefits of intersectoral collaboration in health promotion (38).

The strength of this article includes its innovative aspect regarding the use of the Guide in an intersectoral educational intervention with professionals who work with children. The method applied created space for the interviewees to explain their opinions and experiences in detail, one year after the training experience, allowing a deeper understanding of the phenomena studied, retention of knowledge and progress in professional practices.

The primary limitation of this article is the absence or refusal of many intervention participants to be interviewed, which means that the data presented is not universal, characterizing selection bias. The second limitation is inherent to the method used, as semi-structured interviews intentionally direct participants to what is being investigated, and the way the conversation is conducted is under the command of the interviewer (22,39). In order to mitigate these biases, the interviewer in this study was extensively trained, the script was carefully prepared to allow reflexivity and all analytical processes were carried out in duplicate (22).

More than ten years after the publication of the Guide, despite the various resources, such as handouts, booklets, folders and books, available in the Ministry of Health’s Virtual Health Library, there is still a need to expand the dissemination of the Guide, especially among professionals who are not nutritionists. Efforts towards an intersectoral approach and ongoing health education have the potential to address the problem of childhood obesity and the multiple determinants that affect children’s diet and health (30,38). 

## Data Availability

Not applicable.
