# Supplementary material for: Application of the Food Guide for the Brazilian Population as a training instrument for intersectoral actions: perceptions of professionals in a Brazilian metropolis
Source: Epidemiol Serv Saude. 2025 May 23;34:e20240397. doi: 10.1590/S2237-96222025v34e20240397.en (PMC12105843; doi:10.1590/S2237-96222025v34e20240397.en)
Supplement: Supplementary file 1 [file 2237-9622-ress-34-e20240397-supp01.pdf]

Tabela suplementar 1. Criação e alocação de rótulos nas transcrições das entrevistas, Campinas. São Paulo, 2023

**Criação de rótulo**	
**Exemplo de transcrição**	**Código**
**Caso 5**<br>Foi boa, foi uma troca, né? É, é claro que entre o pessoal da área da saúde: pediatra, nutricionista... A gente tinha uma fala, é... Mais parecida, né? Porém, tinha algumas pessoas que não eram da saúde, é... Né, um psicólogo, é, tinham professores e monitores também de escola... Então, é, não houve conflito em momento nenhum, foi sempre troca, né?	Capacitação enriquecedora
**Caso 8**<br>Propõe um trabalho interdisciplinar, né? De vários saberes, pra tentar assistir a pessoa da forma mais integral possível.	Todo mundo oferece alguma coisa
**Caso 4**<br>Pra... Pra construção do entendimento do que foi explanado pelas nutricionistas.	Dinâmicas utilizadas
**Caso 5**<br>Eu não tinha efetivamente a ideia do que que era um processado, um ultraprocessado, o minimamente processado, entendeu?	Relação ao Guia/conhecimento do Guia
**Caso 6**<br>A dificuldade, qual que é? Porque (...) eu vejo assim, por exemplo, quando a gente fala... A gente foi fazer uma vez em escola, e levamos essa maquete, a gente pergunta: "Quem já comeu esse macarrão instantâneo?" Todo mundo levanta a mão, porque já comeu. Eu acho que, por exemplo, o que que é difícil? Você... A gente trabalha pra nossa realidade. A gente tem uma alimentação exemplar dentro da escola, mas do portão pra fora é muito difícil, eu acho que atingir os pais...	Ambientes alimentares
