# Supplementary material for: Application of the Food Guide for the Brazilian Population as a training instrument for intersectoral actions: perceptions of professionals in a Brazilian metropolis
Source: Epidemiol Serv Saude. 2025 May 23;34:e20240397. doi: 10.1590/S2237-96222025v34e20240397.en (PMC12105843; doi:10.1590/S2237-96222025v34e20240397.en)
Supplement: Supplementary file 2 [file 2237-9622-ress-34-e20240397-supp02.pdf]

Tabela suplementar 2. Excertos da terceira etapa da análise temática: extração e organização dos dados em matrizes conforme os códigos formulados para análise qualitativa. Campinas, São Paulo, 2023

Transcrição	Tema
**Caso 1**<br>Eu acho bem importante no sentido da gente conseguir justamente por meio desse conceito conscientizar que o nosso problema 'é muito mais embaixo'. Então, assim, porque a gente foca bastante, exatamente na criança, em comer em companhia, estímulo, "tudo", só que os exemplos que a criança tem, não são exemplos positivos e não são exemplos saudáveis, então é uma base de ter que pegar isso a um conceito importante, mas justamente para a família entender isso, que a família, os amigos, a escola onde for, não adianta a gente orientar, fazer tudo e não, Entrevistada: (...) coloca a criança para comer junto, faz tudo e aí querer que a criança se alimente de forma saudável, se toda família, se toda, um ambiente escolar, não é propício pra isso, não tá sendo, não tá favorecendo que a criança siga um exemplo positivo, e sim siga um exemplo negativo.	Comensalidade
**Caso 3**<br>Entrevistadora: E você comentou sobre a comensalidade, né, agora. Então, acredito que você tenha conhecimento sobre esse conceito e foi algo que foi apresentado na capacitação, né? Que essa ideia é ligada ao ato de comer em companhia, de atenção ao momento da refeição... Então, queria ver... ouvir um pouco de você, o que você acha desse conceito? E como ele impacta na sua prática profissional?<br>Entrevistada: É engraçado porque, assim, hoje, né? Eu... Eu trabalho com projeto de hortas, né? Então... Não é exatamente comer, mas é o preparar para comer, desde lá do plantio, né? Então, é… Eu acho que tem muita potencialidade, é... A discussão do sistema alimentar, de todos os momentos, né, da alimentação, com relação à coletivização mesmo. Então a utilização dessas, é... De... Dessas ações que são, é... Enfim, todo mundo, né? Come. Assim, é uma questão de sobrevivência pra você ter ferramentas de discussão da organização coletiva, né? Da forma como, enfim, dos projetos de vidas das pessoas, né? Que às vezes... Como eu sempre comento, né, que, é... A prática por exemplo do plantio, né? Você tem uma ferramenta ali, é... De, é... Manutenção da esperança, né? Porque você planta, dali há pouco tempo você colhe e quando você tá trabalhando com... Com comunidades vulneráveis que o sonho e a esperança ela tem uma outra realidade, né? Ela é muitas vezes... Ela é muito imediata, né? É o hoje pra amanhã, é... Ela possibilita isso e eu acho que um aspecto da... Da alimentação, da preparação culinária, do comer junto, né? Também tem muito essa ferramenta, é... De você, é... Poder agregar, né? E, enfim, comemorar, né? Eu acho que, é... Assim, é um tipo de ferramenta educativa, assim, que não tem erro, sabe? Pra qualquer outro tema, enfim...	Comensalidade
**Caso 5**<br>Ah, comer em companhia, né? Pra mim entra aí e... A percepção de educação alimentar e nutricional, então, de uma oportunidade, né?<br>Então pra mim esse... A comensalidade, né? Que é esse momento de, é... de atendimento a uma demanda física, né, muitas vezes é... É social, é... Entra também esse conceito de oportunidade. De se trabalhar a educação alimentar e nutricional e melhorar qualidade, hábito de vida, né? É... Hábito, é... alimentar mesmo, das crianças. E depois, né, isso aí vai ser progressivo para os maiores e depois pros alunos do médio. Então, eles... Eu penso assim, que eles... Eles estão na rede desde o início, né? Então, se a gente já tem isso ali com os "pequenininhos", a gente vai ter como consequência um adolescente que se alimenta bem, depois um adulto que tem bons hábitos... É esse conceito que eu tenho.	Comensalidade
**Caso 8**<br>Bom, no mínimo (risos), o primeiro benefício básico é relações, né? Manter relações, estar com as pessoas, é... Participar ali do ato, então entender "o que eu tô comendo?"; "Quem que tá comigo?"; "Para quem que eu preparo?", né? Então, afetividade, é... preparar algo para alguém... E aí, uma série de outras coisas, né? A gente passa (...) pelo trabalho de fala, né? Então as pessoas vai começando a nomear as coisas, né? Conceito, e estimular para a questão do comer... "O que que eu tô comendo?", né? Experimentar as coisas, enfim... Da onde veio, as histórias de família… Tem uma série de coisas, né? É muito rico e bastante importante, assim, na minha opinião.	Comensalidade
