# Supplementary material for: Application of the Food Guide for the Brazilian Population as a training instrument for intersectoral actions: perceptions of professionals in a Brazilian metropolis
Source: Epidemiol Serv Saude. 2025 May 23;34:e20240397. doi: 10.1590/S2237-96222025v34e20240397.en (PMC12105843; doi:10.1590/S2237-96222025v34e20240397.en)
Supplement: Supplementary file 3 [file 2237-9622-ress-34-e20240397-supp03.pdf]

Protocolo de intervenção

Capacitação - Guia Alimentar para a População Brasileira

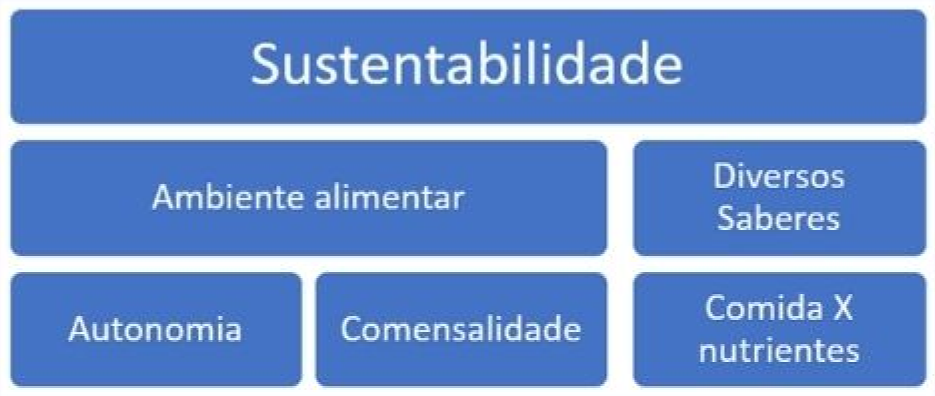

Módulo 1

Introdução ao Guia e atividades de Educação Alimentar e Nutricional. Construção coletiva do conceito de alimentação saudável para crianças

Módulo 2

Abordagem dos princípios do Guia focando na prevenção da obesidade infantil

Módulo 3

Análise do panorama epidemiológico de crianças de dois a cinco anos

Módulo 4

Desenvolvimento de um plano de trabalho aplicado aos temas abordados

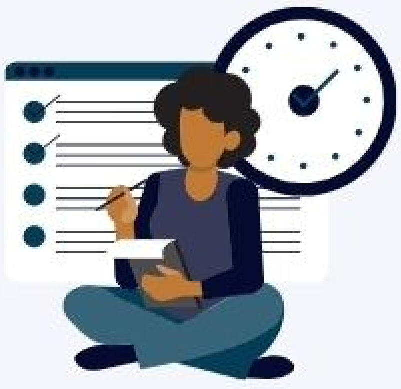

-Princípios do Guia
-Classificação NOVA
-Obesidade Infantil
- Desafios ou barreiras
-Oportunidades
-Materiais Educativos

Foco intersetorial

Planejamento para reunir profissionais (região Noroeste) de áreas interligadas e facilitar a comunicação.

Reuniões

Os encontros foram híbridos, promovendo interação entre os profissionais participantes.

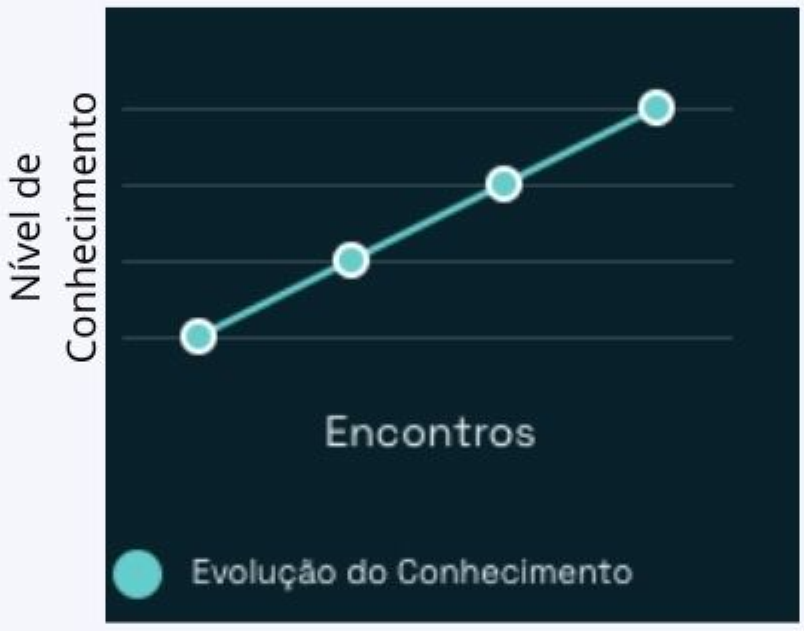

Métodos

O trabalho adaptado e validado em oficina de escuta resultou na criação de um Manual Técnico para facilitar a replicação das oficinas, colaborando para a promoção da alimentação adequada e saudável e prevenção da obesidade infantil.
