# Supplementary material for: Application of the Food Guide for the Brazilian Population as a training instrument for intersectoral actions: perceptions of professionals in a Brazilian metropolis
Source: Epidemiol Serv Saude. 2025 May 23;34:e20240397. doi: 10.1590/S2237-96222025v34e20240397.en (PMC12105843; doi:10.1590/S2237-96222025v34e20240397.en)
Supplement: Translation

# Translation: Aplicação do *Guia Alimentar para a População Brasileira* como instrumento de capacitação para ações intersetoriais: percepções de profissionais em uma metrópole brasileira

**DOI:** 10.1590/S2237-96222025v34e20240397.pt

**Authors:** Vanessa Fernandes Davies, Luiza Navarro de Azevedo, Roberta Camila de Lima Pinheiro, Larissa Galastri Baraldi

**Affiliations:** 1 Universidade Estadual de Campinas, Núcleo de Estudos e Pesquisas em Alimentação, Campinas, SP, Brasil; 2 Universidade de São Paulo, Faculdade de Saúde Pública, São Paulo, SP, Brasil

**Keywords:** Colaboração Intersetorial, Guias Alimentares, Entrevista, Capacitação Profissional, Crianças

## Abstract

**Objetivo:**

Analisar as percepções provindas de uma intervenção educativa fundamentada no *Guia Alimentar para a População Brasileira*, direcionada a servidores públicos que atuam com a população infantil.

**Métodos:**

Estudo qualitativo realizado com nove participantes de uma capacitação profissional, utilizando entrevistas semiestruturadas. As entrevistas ocorreram um ano após a capacitação, em plataforma online, com duração média de 30 minutos. A entrevista foi previamente validada, incluindo perguntas para explorar as percepções sobre a capacitação, a aplicabilidade do Guia e os desafios enfrentados. A análise temática foi realizada em etapas: familiarização com os dados, rotulagem direta, extração e organização dos dados em matrizes, desenvolvimento de temas preliminares e revisão para definição dos temas finais.

**Resultados:**

As entrevistas foram realizadas com 9 pessoas do sexo feminino. A partir da análise dos dados, definiram-se os seguintes temas: Capacitação e enriquecimento profissional; Reflexões produzidas a partir do Guia; e Integração e colaboração intersetorial.

**Conclusões:**

O *Guia Alimentar para a População Brasileira* promoveu reflexões sobre desafios na implementação de ações para prevenção da obesidade infantil.

**Aspectos éticos**

Comitê de ética em pesquisa: Universidade Estadual de Campinas

Número do parecer: 4,924,797

Data de aprovação: 25/8/2021

Certificado de apresentação de apreciação ética: 42713521.8.0000.5404

Registro do consentimento livre e esclarecido: Obtido de todos os participantes antes da coleta

## Introdução

Nos anos 2010 , transformações globais (1) têm impactado a alimentação das populações (2,3), impulsionando, em todas as faixas etárias, as condições crônicas de saúde, em especial a obesidade, o que desafia o sistema de saúde (4-6). A análise das políticas públicas para a população infantil mostra que o enfrentamento do sobrepeso e da obesidade deve passar por controle da publicidade e acordo com a indústria de alimentos; pela superação do ambiente obesogênico; e pela implementação de medidas que promovam a alimentação escolar saudável, por meio de acesso a alimentos da agricultura familiar e educação alimentar e nutricional (7). A ausência de abordagens integradas e intersetoriais é uma barreira para a melhora do quadro nutricional infantil, o que torna essencial a qualificação profissional para atuar nessa perspectiva (6-8).

O *Guia Alimentar para a População Brasileira* é um documento essencial na prevenção da obesidade, pois valoriza práticas alimentares culturais e regionais; é também um importante referencial para diversas áreas (9), fortalecendo o planejamento intersetorial (6,10). Seus princípios destacam que “Recomendações sobre alimentação devem estar em sintonia com seu tempo”, enfatizando a necessidade de uma visão ampla sobre saúde e alimentação para mitigar os impactos negativos do sistema alimentar atual no Brasil, visando um modelo mais sustentável. Após dez anos do seu lançamento, O Guia tornou-se um marco nacional e internacional (10,11), propondo um novo paradigma da nutrição que considera a alimentação em sua relação com os sistemas agroalimentares e a cultura, para além da simples ingestão de nutrientes (12-14).

Tratando-se do público infantil, o documento pode pautar, na área da saúde, o atendimento clínico, e desdobrar-se em orientações específicas para essa fase da vida (7,15). No ambiente escolar, os conteúdos do Guia devem ser integrados às práticas pedagógicas e às ações de educação alimentar e nutricional, para fomentar a autonomia e a consciência crítica sobre a alimentação dos escolares, tornando-se um referencial para toda a comunidade, incluindo pais e educadores (16). Os novos parâmetros para compras da alimentação escolar vêm promovendo a aquisição de alimentos in natura e minimamente processados, em detrimento dos ultraprocessados, pois incorporam a classificação NOVA de alimentos do Guia e promovem um padrão de consumo mais saudável entre os escolares (17). E, no que diz respeito à colaboração intersetorial entre as áreas de Educação e Saúde, institucionalizada pelo Programa Nacional de Alimentação Escolar e pela Política Nacional de Alimentação e Nutrição, é essencial trabalhar conjuntamente para formar hábitos alimentares que respeitem a diversidade e a soberania alimentar (7,18,19). 

Este estudo parte da hipótese de que o Guia oferece conteúdos relevantes para diversos profissionais que atuam com o público infantil, podendo aprimorar práticas de trabalho e fomentar reflexões sobre fatores que afetam a promoção de uma alimentação adequada e saudável. Buscou-se analisar as percepções de profissionais da saúde e educação, envolvidos em uma intervenção educativa sobre o conteúdo do Guia, com foco nas práticas diárias, nos facilitadores e desafios na implementação de ações de prevenção da obesidade infantil e em como os profissionais absorveram os conceitos do Guia.

## Métodos

### Tipo de estudo

Estudo qualitativo que possibilitou compreender as percepções e experiências acumuladas dos participantes, explorando a aplicabilidade do Guia na formação de profissionais de saúde e educação. Alinhando-se à hipótese, aos objetivos e à técnica de coleta, a abordagem teórico-metodológica pragmática permitiu uma exploração livre dos dados, com análise indutiva das falas, para construir os achados apresentados (20,21).

### Descrição da intervenção educativa 

A intervenção educativa integrou o projeto “Ações Intersetoriais para Prevenção da Obesidade Infantil” – uma parceria entre a Prefeitura de Campinas, o Núcleo de Estudos e Pesquisas em Alimentação da Universidade Estadual de Campinas e o Ministério da Saúde. Foram oferecidas oficinas, totalizando 16 horas de capacitação, divididas em quatro módulos, com atividades adaptadas e validadas para o público infantil e o contexto intersetorial (22,23). O conteúdo foi destinado a profissionais de saúde, educação e assistência social, abordando os conteúdos principais do Guia, com foco na prevenção da obesidade infantil e na promoção de uma alimentação saudável. As atividades, realizadas entre fevereiro e março de 2022, ocorreram de forma híbrida, com encontros virtuais e presenciais semanais, conduzidos por facilitadores e baseados na metodologia crítico-reflexiva (24). A capacitação resultou em um manual técnico do Ministério da Saúde para futuras oficinas e incluiu temas como: centralidade do Guia na educação alimentar e nutricional; princípios do Guia e alimentação saudável; prevenção da obesidade; classificação NOVA; ambiente alimentar e comensalidade; desafios na promoção da saúde infantil; e desenvolvimento de plano de trabalho. Mais detalhes dessa intervenção se encontram na Figura suplementar 1.

### Seleção e caracterização da amostra

Amostra intencional composta por profissionais que estiveram presentes na capacitação. O número de participantes variou de 27, no módulo on-line, a 13, no último módulo presencial. Todos os 27 participantes foram convidados a avaliar a intervenção por e-mail e telefone, em pelo menos três ocasiões diferentes, com variação de dia e horário. Ao final, nove profissionais de saúde e educação infantil concederam entrevista; três relataram indisponibilidade; e os demais não responderam ao contato.

### Coleta de dados

Foram realizadas entrevistas semiestruturadas para coletar os dados. Desenvolveu-se um roteiro orientado pela pergunta principal da pesquisa. Cada pergunta do roteiro teve seu próprio objetivo, alinhado à pergunta principal, garantindo a sistematização das informações coletadas e suporte para a entrevistadora, para exploração da questão investigada. A entrevistadora foi treinada para atuar como facilitadora, auxiliando o entrevistado a compreender as perguntas e sentir-se à vontade para expressar sua opinião. Adicionalmente, adotou-se uma postura de acolhimento, atenção e cuidado para com os participantes, a fim de facilitar a fluidez das entrevistas.

O roteiro foi validado pela coordenadora do projeto, na qualidade de especialista, segundo os critérios de coerência com os objetivos, clareza das perguntas, relevância dos temas e abrangência (25). As perguntas seguiram uma sequência crescente de complexidade e incluíram questões de seguimento, para aprofundamento dos temas abordados. A Tabela 1 apresenta em detalhes o roteiro elaborado. 

**Tabela 1:** Roteiro semiestruturado utilizado nas entrevistas realizadas com os profissionais que participaram da capacitação. Campinas, 2023

Pergunta principal	Pergunta de seguimento	Objetivo da pergunta	Nível de complexidade
Me fale o que achou da capacitação.	Se fosse para escolher um assunto dentro da capacitação que você não conhecia e que achou interessante, qual seria? Me diga o que você acha que poderia ser melhorado na capacitação de que você participou.	Conhecer a opinião geral sobre a capacitação.	Pergunta de abertura para iniciar a relação entre entrevistado e entrevistador e, ao mesmo tempo, permitir a expressão de sentimentos e opiniões sobre ajustes para capacitações futuras.
Me conte dos aprendizados que você adquiriu na capacitação; o que você conseguiu colocar em prática.	O que você aprendeu na capacitação que acha difícil de ser colocado em prática? Por quê?	Avaliar como os ensinamentos da capacitação estão no imaginário dos participantes para serem implantados na prática.	Pergunta de nível intermediário, a qual exige que o entrevistado reflita sobre sua aprendizagem na capacitação.
Durante a capacitação, foi apresentado o termo “comensalidade”, que está ligado ao ato de comer em companhia. Me fale o que acha deste conceito.	Após a capacitação, como você pensa a comensalidade, ou o comer em companhia, e a promoção da alimentação saudável?	Entender o que foi aprendido sobre comensalidade, um conceito-chave do *Guia Alimentar para a População Brasileira*.	Pergunta de nível intermediário, mas um pouco mais complexa que a anterior, pois exige do entrevistado buscar o aprendizado sobre um conceito incomum em sua realidade de trabalho.
Não sei se você lembra, mas pessoas de diferentes áreas participaram das oficinas de capacitação. O que você achou desta integração?	Como você vê pessoas de diferentes áreas trabalhando com a promoção da alimentação saudável? E no seu trabalho, como você imagina diferentes profissionais trabalhando com o tema da promoção da alimentação saudável? O que poderia ser feito para integrar pessoas de diferentes áreas para trabalhar o tema da alimentação saudável?	Avaliar o entendimento dos participantes a respeito de ações intersetoriais para a prevenção da obesidade infantil.	Pergunta complexa, uma vez que pode envolver sentimentos relacionados a processos de trabalho, e dinâmicas de trabalho com profissionais de diferentes áreas.
Me fale como você percebe a integração entre profissionais de saúde e educação para trabalhar a promoção da alimentação saudável.	Quais seriam as facilidades que existem para que esta integração possa acontecer? E as dificuldades? O que poderia ser melhorado?	Avaliar a aplicabilidade do que foi abordado na capacitação com relação à consolidação e disseminação de ações de Promoção da Saúde e Alimentação Adequada e Saudável nas práticas intersetoriais dos profissionais de saúde e educação nos seus territórios de abrangência.	Pergunta complexa, pois envolve questões relacionadas a opiniões, reflexões sobre possibilidades e obstáculos a serem enfrentados, assim como a construção de soluções para o problema apresentado.
Para terminar, gostaria de saber se existe algo mais que gostaria de falar sobre a capacitação e que não foi abordado nas perguntas que fizemos a você.	-	Promover a abertura, permitindo que o entrevistado complemente ou aborde algo relevante não explorado na entrevista.	Pergunta final, para garantir ao entrevistado a oportunidade de se expressar.

O contato com os participantes ocorreu um ano após a intervenção, possibilitando avaliar os desdobramentos da intervenção e as experiências retidas na memória dos profissionais. As entrevistas, realizadas em março de 2023, duraram em média 30 minutos e foram gravadas via Google Meet.

### Análise dos dados

As gravações das entrevistas foram carregadas na plataforma PinPoint para transcrição. Foi necessária uma revisão manual para garantir a fidelidade das transcrições, que foram realizadas palavra por palavra, assegurando máxima precisão.

Dois pesquisadores, treinados por um pesquisador sênior, conduziram de forma independente a análise temática dos dados. O método escolhido permite adaptações a diferentes abordagens teóricas, facilita a síntese e descrição de grandes conjuntos de dados, identifica similaridades e diferenças entre entrevistados, gera percepções inesperadas e possibilita interpretações sociais e psicológicas dos dados (26).

O processo analítico constou das seguintes etapas: familiarização com os dados, que incluiu leitura e releitura das transcrições; criação de códigos, com base na pertinência dos extratos à pergunta de pesquisa; codificação direta das entrevistas transcritas (Tabela suplementar 1); extração e organização dos dados em matrizes conforme os códigos (Tabela suplementar 2); análise preliminar para o desenvolvimento de temas; e subsequente revisão, para se verificar a coerência interna e externa dos temas, culminando na definição dos temas finais que sintetizam o conteúdo das entrevistas (26). Um resumo detalhado das etapas de análise está disponível no material suplementar.

Após a análise independente, os pesquisadores se reuniram para comparar e consolidar as análises, discutindo o agrupamento final dos temas. As discordâncias foram debatidas entre os autores deste estudo, para se alcançar um consenso. A base de dados completa, com todos os temas e rótulos criados, encontra-se disponível em repositório de dados virtual (27).

## Resultados

Todas as nove respondentes eram do sexo feminino, com idade média de 43 anos e servidoras públicas há 11 anos, em média. Dentre elas, cinco eram nutricionistas (uma atuante em equipe de Núcleo de Apoio à Saúde da Família e quatro atuantes na alimentação escolar), uma pediatra, uma socióloga atuante em projetos de hortas escolares, uma terapeuta ocupacional atuante em equipe do Núcleo de Apoio à Saúde da Família e uma diretora de escola infantil municipal. 

A partir da análise dos dados, definiram-se os seguintes núcleos temáticos: Capacitação e enriquecimento profissional; Reflexões produzidas a partir do Guia; e Integração e colaboração intersetorial.

A seguir, são apresentados os detalhamentos dos temas oriundos das análises das entrevistas; exemplos de falas das entrevistadas dentro de cada tema encontram-se na Tabela 2. 

**Tabela 2:** Excertos extraídos das falas de entrevistados participantes da intervenção educativa, segundo tema criado. Campinas, 2023

Tema	Citação
**I** **Capacitação e enriquecimento profissional**	“...trouxe um outro olhar sobre como utilizar o Guia como ferramenta... eu me lembro até hoje de um bate-papo que nós estávamos e era uma chuva de ideias: “o que é comida para criança?”... e aí várias pessoas participando – não todas da área da saúde –, colocaram “Danoninho”, a outra “Toddynho”... Então para entender como essa visão, ela é errada, mas ela já vem embutida nas pessoas, e a gente acaba tendo como referência e é isso que a gente vem tentando mudar, com esses trabalhos que estão sendo feitos, então... Abrir os olhos, para o Guia Alimentar?” Caso 6, profissional da educação. “Para a gente é muito fácil ouvir falar do Guia Alimentar... Mas muita gente não tem conhecimento... Mas isso é um trabalho que tem que ser contínuo, não... uma vez só... Eu acho que é uma coisa... Assim, de “bater na mesma tecla” várias vezes, continuamente, para, aí sim, a gente ver uma diferença - às vezes pequena - mas ver uma diferença, né?” Caso 6, profissional da educação. “Eu não tinha efetivamente a ideia do que era um processado, um ultraprocessado, o minimamente processado, entendeu?” Caso 5, profissional da educação. “E o que você gasta em um você consegue comprar muito mais nos alimentos in natura que a gente tem realmente a ideia de que o *in natura* é mais caro que o processado, que o ultraprocessado, né? Falei: então, espera aí, nossa!” Caso 5, profissional da educação. “Estar em contato com outros profissionais, também de outros locais. Não só outros profissionais do mesmo posto de saúde, mas de outros locais também. Acho que... Foi válido para ver um pouco o que era de diferente em cada um dos lugares, em cada um dos setores e as coisas que se assemelhavam, as dificuldades que se assemelhavam nesse sentido.” Caso 1, profissional de saúde. “Eu achei bem interessante, principalmente porque envolveu profissionais de outros... Outras áreas, né? Mas que trabalham com o público que a gente atende também, né? Então isso eu achei muito interessante. É... Foi possível a gente trocar ideias, o pessoal conhecer mais do trabalho da alimentação escolar também. E as dinâmicas utilizadas, né? As atividades utilizadas para construir ações, é... Para a construção do entendimento do que foi explanado pelas nutricionistas, eu achei que foi bem interessante.” Caso 4, profissional da educação. “A gente teve a dinâmica de cortar e colar, de pensar no território... né? Eu não conhecia muitas pessoas ali. Eu... Eu, na verdade, eu fiz contatos ali e desenvolvi depois projetos com essas pessoas – muito – de centro de saúde, eu fui convidada para falar sobre o PNAE para um grupo de residência lá do posto de saúde São Bernardo através de um contato que eu fiz nessa reunião.” Caso 5, profissional da educação. “Cada um tem um ponto de vista, porque cada um tem uma vivência. Seja num posto de saúde ou numa escola, cada... Uma visão diferente, mas o tema era o mesmo. Então eu acho que isso é muito importante… Eu acho… A ligação… E ter mais encontros assim faria diferença, sabe? Porque assim a gente vai entendendo um lado do outro... Porque tudo se comunica no final.” Caso 6, profissional da educação.
II **Reflexões produzidas a partir do** *Guia Alimentar para a População Brasileira*	“Transversalmente, de alguma forma, todo o conteúdo do Guia eu consigo usar... A discussão de sistemas alimentares eu consigo usar, a comensalidade, para organização coletiva, comunitária, é tranquilo de utilizar também... Ah, eu acho que com relação ao conteúdo do Guia eu consigo utilizar tudo.” Caso 1, profissional da saúde. “... A gente fica muito refém da questão de indústria, de mídia, que também foi levantado na capacitação, então o comercial de TV, propaganda, coisa de supermercado... Qualquer lugar que a gente vai... Tentar levar e mostrar para as mães nesse sentido.” Caso 1, profissional de saúde.
**II** **Reflexões produzidas a partir do** *Guia Alimentar para a População Brasileira*	“Então para mim a comensalidade, que é esse momento de muitas vezes social, entra também esse conceito de oportunidade. De se trabalhar a educação alimentar e nutricional e melhorar qualidade, hábito de vida, hábito alimentar mesmo, das crianças. E depois, isso aí vai ser progressivo para os maiores e depois para os alunos do médio. Então, eles... Eu penso assim, que eles... Eles estão na rede desde o início. Então, se a gente já tem isso ali com os pequenininhos, a gente vai ter como consequência um adolescente que se alimenta bem, depois um adulto que tem bons hábitos... É esse conceito que eu tenho”. Caso 3, profissional de saúde. ”Então... Eu cubro três, quatro centros de saúde, qual é o tempo que eu tenho para estar com os meus colegas de trabalho? Qual é o tempo que eu tenho para construir saberes juntos? Entre deslocamento, entre uma agenda de suprir mil coisas, então assim, tem que ter uma proposta de trabalho traçada para que aconteça esses encontros.” Caso 1, profissional da saúde. “Eu acho bem importante no sentido da gente conseguir justamente por meio desse conceito conscientizar que o nosso problema ‘é muito mais embaixo’. Então, assim, porque a gente foca bastante, exatamente na criança, em comer em companhia, estímulo, só que os exemplos que a criança tem não são exemplos positivos e não são exemplos saudáveis, então é uma base de ter que pegar isso a um conceito importante, mas justamente para a família entender isso, que a família, os amigos, a escola onde for, não adianta a gente orientar, fazer tudo... Coloca a criança para comer junto, faz tudo e aí querer que a criança se alimente de forma saudável, se toda família, se toda, um ambiente escolar, não é propício para isso, não tá favorecendo que a criança siga um exemplo.” Caso 1, profissional de saúde. “Eu procuro sempre abordar essa questão da importância do comer à mesa junto da família... Falar sobre o alimento que está sendo servido, do exemplo que o adulto é e da segurança que ele traz para a criança; e a gente percebe, pela carinha dos pais, que quando a gente fala que a criança tem que comer à mesa e não em frente à TV ou ao computador, a reação de que como isso acontece em casa. Que eles não fazem as refeições à mesa, não fazem juntos. E na escola, a gente observa que as crianças comendo junto, uma acaba imitando a outra. Se um não quer, o outro também fala que não quer e o educador insiste para que ele prove; um prova, daí o outro acaba provando também…Então, é de extrema importância esse convívio, esse momento da refeição.” Caso 4, profissional da educação. “Eu sou uma pessoa que gosta de cozinhar, eu cozinho todos os dias, eu não compro coisa fora, eu gosto de cozinhar. Então, assim, faz diferença, mas tudo isso, eu acho que foi uma sementinha plantada lá quando era criança.” Caso 6, profissional da educação. “...Porque, fora o Guia Alimentar, a gente tem o manual da Sociedade Brasileira de Pediatria, que a gente também usa e que os pediatras usam, mais como referência do que o próprio Guia. Mas eles… Eles estão bem alinhados. Assim, tem uma outra coisa, que a gente, infelizmente, vê que é um pouco direcionado, porque existe um patrocínio.” Caso 8, profissional da saúde.
**III** **Integração e colaboração intersetorial**	“Se não tivesse talvez esse espaço facilitador, a gente não teria realmente acessado um ao outro, eu acho que seria muito difícil, é acho que tem que ter sim uma pessoa responsável por promover esse encontro, promover essa integração, com esses profissionais; então, uma pessoa que está ali em contato com todos os equipamentos, que faça essa reunião desses profissionais que estariam dispostos a seguir com ações desse tipo. Então tem que ter um facilitador. Se não tivesse, a gente não teria meios para isso. Acho que até teria, mas teria que elencar um responsável para fazer isso e talvez demorasse muito mais tempo.” Caso 2, profissional da educação. “A gente vê a necessidade das políticas públicas serem planejadas em conjunto, de ser um plano mesmo de política pública integrada. Desafiador, muito desafiador; isso se reflete também na ponta. Mas é uma discussão que a gente fala muito, porque o usuário, ele é uma pessoa só, ele não é uma pessoa fragmentada. Então como que a gente também fragmenta todos os atendimentos?” Caso 3, profissional da saúde.

No tema I, as participantes apontaram a metodologia participativa utilizada durante a capacitação como um fator positivo. Igualmente, o Guia como referencial teórico para a capacitação foi citado como ponto positivo, e foi relatado o desconhecimento do material, não só por parte dos profissionais, mas também pela população em geral. As participantes mostraram inquietação sobre alguns produtos infantis amplamente utilizados no dia a dia, considerados “comida para crianças”, mas que, na realidade, são ultraprocessados; o sentimento expressado apontou como a tradução das recomendações do Guia, na prática, ainda não era contemplada por parte das participantes da oficina. A partir das oficinas, verificou-se a necessidade de divulgar materiais advindos do Guia e elencaram-se os conteúdos que mais chamaram a atenção positivamente e agregaram conteúdo teórico, segundo as profissionais, dentro do material utilizado, a saber: a classificação NOVA de alimentos; o custo dos alimentos in natura em comparação aos ultraprocessados; a aplicação do conceito de comensalidade; o desenvolvimento de habilidades culinárias; e a importância da rotulagem nutricional.

Os demais comentários favoráveis à capacitação estiveram relacionados à relevância do tipo de integração gerada, posto que as atividades em grupo permitiram comparar diferentes pontos de vista técnico-profissionais, possibilitando vislumbrar diversas formas de aplicação do conhecimento adquirido. As profissionais relataram que conhecer as experiências e dificuldades dos diferentes locais de trabalho, dentro do mesmo município, possibilita planejar o enfrentamento, seja de forma conjunta no território ou localmente. Foi apontada a necessidade da adoção permanente de estratégias de capacitação diversificadas que envolvam, por exemplo, a melhor compreensão das realidades e contextos de trabalho dos demais servidores do município. Esse conjunto de práticas colaborativas foi visto como essencial para alcançar um público mais amplo, incluindo pais, educadores e a comunidade do território, visando promover uma abordagem integralizada à saúde infantil.

Alguns pontos não favoráveis da capacitação foram citados, como a necessidade de maior tempo para as oficinas, o não aprofundamento de alguns temas, e a pouca adesão dos profissionais à capacitação, sobretudo dos servidores da educação. 

As reflexões produzidas pelos entrevistados no tema II foram, provavelmente, originadas em decorrência do referencial teórico principal utilizado, o Guia. Algumas entrevistadas relacionaram aspectos do Guia a suas experiências pessoais e de prática profissional. Por exemplo, algumas participantes apontaram que possuem o hábito de cozinhar o próprio alimento, fato que, por sua vez, se relaciona com o ponto tratado no Guia sobre habilidades culinárias; a importância de ser um indivíduo crítico diante da publicidade e propaganda da indústria de alimentos; e o próprio entendimento do conceito de comensalidade para a promoção de hábitos alimentares saudáveis. 

As profissionais também apontaram questões contextuais, como a relevância dos ambientes alimentares na formação e determinação de hábitos alimentares. Discorreram principalmente sobre a dissimilaridade entre o ambiente alimentar doméstico e o escolar, considerando a variedade das refeições escolares, que muitas vezes não é repetida em casa devido à falta de acesso a alimentos diversos. Destacou-se a influência familiar no estabelecimento de hábitos alimentares, sendo enaltecida a participação ativa da família durante as refeições, com a criação de um ambiente propício para a experimentação de novos alimentos. 

A comensalidade foi apontada como agente de inferência direta no momento da refeição e em suas reverberações. A maioria das entrevistadas relatou que sempre abordou essa temática no seu cotidiano de trabalho, apesar de algumas não utilizarem o termo “comensalidade” ou não terem tido conhecimento, previamente à capacitação, da magnitude do conceito de comensalidade. Sugeriu-se o “comer em companhia” como promotor do “comer saudável” e a introdução de novos alimentos para a criança, alinhados com a prática da “degustação pedagógica” durante o período de alimentação na escola, que é a proposta de os educadores se alimentarem juntamente com os alunos como uma forma de educação alimentar e nutricional.

Houve abertura das entrevistadas em utilizar o Guia em seus processos de trabalho. Contudo, informaram a existência de outros materiais corriqueiramente utilizados, como manuais organizados por sociedades médicas. A possibilidade de conflito de interesses, encontrada em materiais de diversas sociedades, foi contraditoriamente citada por profissionais que estão acostumadas a utilizá-los. 

A importância da integração e colaboração intersetorial na promoção da alimentação adequada e saudável foi explorada no tema III, com ênfase na contribuição singular de cada setor para uma abordagem mais abrangente no cuidado infantil. Profissionais da saúde e educação reconheceram unanimemente a necessidade de cooperação mútua para alcançar resultados mais eficazes. A comunicação assertiva e encontros regulares entre equipes multiprofissionais foram fatores identificados como fundamentais para o êxito das iniciativas.

As participantes expressaram a importância da intersetorialidade em seu cotidiano de trabalho, uma vez que as políticas públicas são planejadas sob esta ótica; seguindo a mesma lógica, mencionou-se que o público atendido não pode ser tratado de forma fragmentada, trazendo a perspectiva da integralidade do cuidado.

Como resultado positivo da capacitação, as entrevistadas pontuaram avanços na comunicação entre as profissionais. Em particular, pode ser apontada a criação de um grupo no aplicativo WhatsApp entre as próprias integrantes, o que se entendeu como um facilitador de comunicação, tornando-a mais constante e organizada entre essas profissionais da saúde e da educação. É importante que se destaque essa melhoria, dado que, anteriormente à capacitação, essas servidoras – embora atuassem em uma mesma região do município – não se conheciam e não dispunham de um canal eficiente para diálogo. A implementação dessa ferramenta de comunicação contribuiu para o fortalecimento da rede profissional e para o aprimoramento de contato entre o grupo, o que permitiu a organização de novos encontros independentes da capacitação, se tornando um potencial indutor de ações de alimentação e nutrição entre essas servidoras. A importância de um espaço (como o que foi concedido durante a capacitação) e de um líder para organizar os momentos de interação profissional foi mencionada como algo que facilitaria as trocas e atividades para promoção de uma alimentação adequada e saudável, frente à grande demanda que as profissionais já possuem no cotidiano de trabalho.

Os desafios na implementação de ações intersetoriais foram evidenciados pela falta de comunicação entre setores, sobrecarga de trabalho em equipes reduzidas, resistência a mudanças necessárias para a educação alimentar e nutricional, além das limitações em recursos humanos adequados e comprometidos. Esses obstáculos dificultam, segundo as entrevistadas, a construção de relações colaborativas e a realização de ações conjuntas, impactando a eficácia das intervenções em saúde e prevenção de doenças. A persistência e a continuidade das ações foram apontadas como essenciais para enfrentar os desafios supracitados.

Apesar dos desafios mencionados, a intersetorialidade foi reconhecida como essencial para oferecer cuidado integral e para a compreensão das necessidades dos pacientes de forma mais ampla. Em síntese, a colaboração intersetorial emergiu como uma abordagem crucial para enfrentar os desafios na promoção da saúde e alimentação saudável, demandando comprometimento, persistência e uma alocação adequada de recursos para seu sucesso.

## Discussão

A intervenção educativa fundamentada no Guia teve boa receptividade e observou-se uma abertura para a utilização das diretrizes do Guia, para o compartilhamento de práticas de trabalho e para novas oportunidades de interação intersetorial. 

A implementação do Guia contribui para essa organização, ao promover uma abordagem integral de saúde, visando à mudança nos hábitos alimentares e à redução do consumo de ultraprocessados, o que pode ajudar na prevenção e controle da obesidade (28,29). A distribuição de materiais educativos e o treinamento de facilitadores são as formas mais comuns de implementação dos guias alimentares, podendo impactar positivamente a demanda por políticas públicas, à medida que a apropriação das recomendações pela população potencializa a superação de obstáculos sistêmicos. Contudo, a realização desse processo requer o engajamento dos setores de educação e saúde, o que frequentemente se revela desafiador, devido à falta de alinhamento de agendas, conhecimento ou interesse, conforme evidenciado neste estudo (29). A experiência de aplicabilidade do Guia na capacitação de profissionais de duas áreas-chave na promoção da alimentação adequada e saudável é uma contribuição deste estudo. O estímulo de reflexões sobre temáticas não abordadas nas formações tradicionais dos profissionais, que não nutricionistas, pode proporcionar aumento na confiança individual e coletiva para aplicação prática dos conteúdos trabalhados na capacitação (30). Parte da aceitação em adotar o Guia como um material oficial para embasamento das práticas profissionais deve-se ao fato de este ter se revelado como um material de apoio multidisciplinar, que dialoga com diferentes aspectos da alimentação que fazem parte do dia a dia de todos os indivíduos, o que despertou o interesse das participantes. Isso corrobora a afirmação de que o Guia brasileiro cumpre os objetivos e metas esperados de um guia alimentar de qualidade, sendo um material fundamentado em evidências científicas, prático, de fácil compreensão e adaptável a diferentes grupos etários (31,32).

As reflexões produzidas sobre a comensalidade entre as participantes representaram um achado positivo, uma vez que os aspectos abordados nessa dimensão – desde o modo de comer e a presença de companhia à mesa, até o tempo e a atenção destinados à refeição – podem ser considerados determinantes para uma alimentação saudável (33). A adoção do conceito de comensalidade é um dos pontos inovadores do Guia e contribui na mudança de abordagens nutricêntricas e reducionistas para uma forma mais holística e sustentável de trabalhar a alimentação (34,35). Nota-se que as diversas dimensões do Guia, sobretudo aquelas que não tratam diretamente das recomendações dietéticas e nutricionais, são as que mais aproximam as profissionais que não possuem formação em nutrição.

 Embora as participantes da capacitação tenham demonstrado disposição para integrar o conteúdo do Guia em seus processos de trabalho, um alerta foi levantado por uma das participantes sobre a tendência dos profissionais de saúde em utilizar materiais de referência de organizações e sociedades de diversas especialidades médicas, que podem apresentar viés devido a parcerias com empresas com conflitos de interesse. Para capacitações futuras, sugere-se uma comparação mais direta entre esses materiais frequentemente produzidos e o conteúdo do Guia, o que poderia fomentar reflexões críticas sobre a influência das indústria de alimentos e farmacêutica na elaboração de materiais e nas práticas de saúde. O conflito de interesse tem sido amplamente discutido na relação entre acadêmicos e o financiamento da produção de evidências científicas, mas pode não ser uma preocupação plenamente reconhecida entre os profissionais de saúde (36). O uso do Guia para abordar essa questão é relevante, pois se trata de um recurso baseado em evidências científicas e isento de interesses econômicos.

A criação de um grupo de comunicação permanente, por meio do aplicativo WhatsApp, para a troca de experiências entre as participantes deste estudo, pode ter sido facilitada pela metodologia empregada nas oficinas, que favoreceu a aproximação entre as servidoras. A abordagem dialógica, particularmente a de Paulo Freire, incentiva o diálogo e a reflexão crítica, promovendo um ambiente propício para a construção de vínculos, formação de redes e colaboração contínua entre educadores (25). O grupo formado constituiu um primeiro passo para superar barreiras intersetoriais, considerando as divisões geográficas em grandes cidades, como Campinas, e a ausência de meios que permitam a comunicação entre profissionais de diferentes áreas. Mais pesquisas são necessárias sobre o papel das tecnologias, como aparelhos celulares, para facilitar a colaboração profissional, superando limitações de acesso físico e tempo (37). Conforme ressaltado pelas entrevistadas, é fundamental o investimento em recursos financeiros e humanos para sustentar e maximizar os benefícios da colaboração intersetorial na promoção da saúde (38).

O ponto forte deste artigo inclui seu aspecto de inovação no que diz respeito à utilização do Guia em uma intervenção educativa intersetorial com profissionais que trabalham com a população infantil. O método aplicado gerou espaço para as entrevistadas explicarem suas opiniões e experiências em detalhes, um ano após a experiência vivida da capacitação, permitindo uma compreensão mais profunda dos fenômenos estudados, retenção de conhecimento e avanços nas práticas profissionais.

O primeiro ponto limitante é a ausência ou recusa de muitos participantes da intervenção em conceder a entrevista, o que faz com que os dados apresentados não sejam universais, caracterizando viés de seleção. A segunda limitação é inerente ao método utilizado, pois entrevistas semiestruturadas direcionam intencionalmente os participantes para o que está sendo investigado, e a condução da conversa está sob comando do entrevistador (22,39). Para mitigar esses vieses, treinou-se extensivamente a entrevistadora deste estudo, preparou-se cuidadosamente o roteiro para que permitisse reflexividade e fizeram-se todos os processos analíticos em duplicata (22).

Passados mais de dez anos desde a publicação do Guia, apesar dos diversos recursos, como apostilas, livretos, fôlderes e livros, disponíveis na Biblioteca Virtual em Saúde do Ministério da Saúde, ainda persiste a necessidade de ampliar a divulgação deste documento, especialmente entre profissionais não nutricionistas. Esforços para a abordagem intersetorial e a educação permanente em saúde têm o potencial de enfrentar o problema da obesidade infantil e os múltiplos determinantes que afetam a alimentação e a saúde das crianças (30,38). 

## Data Availability

Não se aplica.
